# Detection of Two Species of the Genus Parapoxvirus (Bovine Papular Stomatitis Virus and Pseudocowpox Virus) in Ticks Infesting Cattle in Burkina Faso

**DOI:** 10.3390/microorganisms8050644

**Published:** 2020-04-28

**Authors:** Achille Ouedraogo, Léa Luciani, Olivier Zannou, Abel Biguezoton, Laura Pezzi, Laurence Thirion, Adrien Belem, Claude Saegerman, Rémi Charrel, Laetitia Lempereur

**Affiliations:** 1 Laboratory of Parasitology and Parasitic Diseases, Center for Fundamental and Applied Research for Animal and Health (FARAH), Faculty of Veterinary Medicine, ULiège, 4000 Liège, Belgium; 2 Unité Maladies à Vecteur et Biodiversité (UMaVeB), Centre International de Recherche-Développement sur l’Élevage en zone Subhumide (CIRDES), Bobo-Dioulasso 01, Burkina Faso; 3 Unité des Virus Émergents (UVE: Aix Marseille Univ, IRD 190, INSERM U1207, IHU Méditerranée Infection), 13005 Marseille, France; 4 Research Unit in Epidemiology and Risk Analysis Applied to Veterinary Sciences (UREAR-ULiège), Center for Fundamental and Applied Research for Animal and Health (FARAH), Faculty of Veterinary Medicine, ULiège, 4000 Liège, Belgium; 5 Université de Corse Pascal Paoli, Laboratory of Virology, UR7310 BIOSCOPE, 20250 Corte, France; 6 Laboratoire de Santé Animale Tropicale, Institut du Développement Rural, Université Nazi BONI, Bobo-Dioulasso 01, Burkina Faso

**Keywords:** bovine papular stomatitis, pseudocowpox, virus, arbovirus, poxviridae, cattle ticks, West Africa, Burkina Faso

## Abstract

The molecular identification of arboviruses in West Africa is of particular interest, due to their zoonotic potential in a population living in close contact with livestock, and in a region where the livestock migration across borders raises the risk of diseases infection and dissemination. The aim of the study was the screening of potential circulating arboviruses and the assessment of their zoonotic implications. Therefore, ticks were collected on cattle located in three provinces of eastern Burkina Faso. Tick pools were tested using a panel of genus-specific real-time assays targeting conserved regions of parapoxvirus, orthopoxvirus, flavivirus and phlebovirus. On the 26 farms visited, a total of 663 ticks were collected. Four genera and six tick species were morphologically identified, with *Amblyomma variegatum* and *Hyalomma* spp. being the most represented species. No arboviruses were found. However, this study highlights the presence of pseudocowpox virus (8.2%) and bovine papular stomatitis virus (5.8%) among the positive tick pools. BPSV positive ticks were found in herds sharing water and pastures resources and with a history of seasonal transhumance. Therefore, common grazing and the seasonal transhumance are likely to support the transmission of the virus. This could have important health and economic impacts, especially regarding transboundary cattle movements.

## 1. Introduction

In West Africa, cattle farming is of great importance, as it generates income for a large part of the population [1]. However, infectious diseases represent an important constraint by hindering production and productivity, causing huge economic losses. Additionally, the zoonotic impact of these diseases is often neglected, especially in African countries. Vectors are able to transmit a wide range of pathogens, including parasites, bacteria and viruses, particularly an arthropod borne virus group named “arbovirus”. A panel of arboviruses, mainly including families of flaviridae and bunyaviridae, are highly present in Africa. In the family of flaviviridae, most of the species belonging to the flavivirus genus are zoonotic arboviruses [2]. They are transmitted between vertebrate hosts by mosquitoes or ticks across a wide range of geographical distribution. In Africa, most of these viruses are of particular medical importance, with dengue, yellow fever and zika viruses being significant public health threats [3]. Additionally, virulent strains of West Nile virus originating from Africa emerged, especially in Europe and the United States, causing viral encephalitis in humans, horses, camelids, and birds [2,3,4]. The family of bunyaviridae also includes important zoonotic arboviruses such as the Rift Valley fever virus (RVFV) belonging to the genus phlebovirus, and the Crimean-Congo hemorrhagic fever virus (CCHFV), belonging to nairovirus. RVFV can cause severe diseases in both humans and animals, resulting in significant economic losses due to death and abortion, especially in livestock animals [4]. CCHFV is an emerging problem in many parts of the world [5]. The main vector is represented by *Hyalomma* tick species infesting wild and domestic ruminants. This disease could affect humans; primarily farmers, veterinarians and others coming in contact with livestock and infected ticks [4,6,7]. Despite their harmful effects, arboviruses and other viruses in general have received limited research attention in West Africa. In addition to the arboviruses, the family of poxviridae, mainly represented by the genera parapoxvirus and orthopoxvirus, includes viruses affecting livestock animals and humans such as the pseudocowpox virus (PCPV) and the bovine papular stomatitis virus (BPSV). Commonly, they cause mild diseases in cattle, although they are able to induce a significant loss of productivity [8,9]. Occasionally, humans can be infected through direct contact with the lesions of infected animals. Clinical manifestations are observed on hands, and they are thus presented as occupational zoonotic diseases [10]. Besides the direct transmission, other viruses of this family could also be transmitted throughout vectors such as the agent of the lumpy skin disease. This disease is well known on the African continent, where it is transmitted by flies and ticks [10,11].

Disease surveillance is often neglected, especially in animals, mainly due to the limited resources and presently, for security reasons, due to armed conflicts. Therefore, using the tick species harbored by domestic animals as virus sentinels is a convenient and cost-effective manner for monitoring the circulation of potential pathogens in this region. The aim of this study is to provide information on arboviruses circulating in ticks infecting cattle in eastern Burkina Faso. Here, their molecular identification is of particular interest, due to their zoonotic potential in populations living in close contact with livestock, and where the traditional livestock migration across borders raises the risk of diseases’ infection and dissemination.

## 2. Materials and Methods

### 2.1. Ticks Collection and Morphological Identification

**Figure 1:**
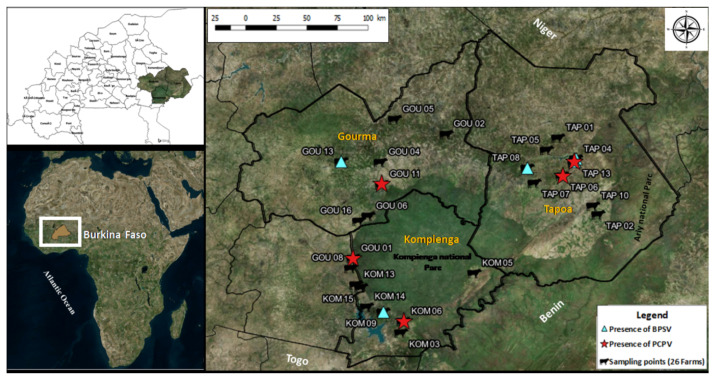
Localization of tick collection and positive pools of ticks in the study area (Eastern Burkina Faso) Gou: Gourma; Kom: Kompienga; Tap: Tapoa.

Ticks were collected from July to August 2017 on zebu cattle (Bos indicus) located in the provinces of Gourma (12°03’41.65”N, 0°21’30.35”E), Kompienga (11°24’59.99”N, 0°54’59.99”E) and Tapoa (12°14’58.95”N, 1°40’33.85”E), in eastern Burkina Faso (Figure 1). Herds were randomly selected among a list of volunteers in each province. The inclusion criteria at the herd level were the minimum size of 50 heads per herd and the minimal distance of 2 km separating contiguous herds. The cattle of both sexes were selected and classified in two groups according to their age: 3– to 12–months-old (young) and over 12-months-old (adult). The identification code was attributed to each cattle, and each sampling point was characterized by the name of the locality and GPS coordinates. The whole skin of animals was inspected, and ticks were collected manually. Ticks were stored in collection jars with lids previously drilled and closed with compress, in order to allow their survival until returning to the laboratory. Containers were then placed in a plastic bin, with a damp mop on the bottom. Once in the lab, ticks were sorted and only live specimens were used in the study. Moreover, farm owners were asked to provide information on the health status of the animals, through a standardized questionnaire. At the laboratory, ticks were identified at species level at room temperature under stereomicroscope, using an identification key [12] and immediately stored at −20 °C.

### 2.2. Nucleic Acids Extraction

Ticks belonging to the same species and collected from the same animal were pooled together. Pools of ticks were crushed using the mixer millMM400 (RETSCH^®^, Haan, Germany) in HBSS (Hanks’s BalancedSalt Solution, ThermoFisher, USA) at 30 cycles/s for 30 min, followed by centrifugation at 6160*g* for 10 min. Total nucleic acid extraction was performed on a QIAcube HT (Qiagen^®^, Venlo, The Netherlands), using a QIAamp Cador Pathogen kit, according to the manufacturer’s instructions. Eluates were stored at −20 °C, while a part of the crushed pools was stored at −80 °C.

### 2.3. Real-Time PCR

Tick pools were tested using a panel of genus-specific real-time assays targeting conserved regions of parapoxvirus, orthopoxvirus, flavivirus and phlebovirus (Table 1). Positive PCR samples (ct < 35) to these genus-specific assays were submitted to species-specific real-time PCR, targeting highly conserved gene sequences (Table 1). Among parapoxvirus, ORF virus, PCPV and BPSV have been tested [13]. In addition, RVFV [14], and CCHF [15] were tested using a specific assay. Molecular assays were performed with SuperScript III Platinum One-Step qPCR kit (Invitrogen-Thermo Fisher Scientific^®^, Waltham, Ma, USA) in a BioRad CFX96 thermal cycler, software version 3.1 (BioRad Laboratories, Irvine, Ca, USA). Sequencing was subsequently applied to all positive samples (ct < 35) to both generic and specific PCR, using next generation sequencing (Ion Torrent, Life Technologies and CLC Genomics Workbench software, Waltham, Ma, USA). Primers used for the sequencing were those targeting the B2L gene [16]. Detection rates of DNA viruses were compared using Fisher exact test (*p* < 0.05). The data analysis was conducted using the R statistical software (version 3.6.1).

### 2.4. Phylogenetic Analysis

After a blast search (https://blast.ncbi.nlm.nih.gov/Blast.cgi), sequences were aligned using Mega_X_10.1.7 (https://www.megasoftware.net/). Thereafter, a neighbor-joining phylogenetic tree was generated. The percentage of bootstraps were calculated for 100 replicates.

## 3. Results

### 3.1. Ticks Collection and Identification

Of the 26 farms visited (9 in Gourma, 7 in Kompienga and 10 in Tapoa), a total of 663 ticks were collected on 102 cattle inspected (15 in Gourma; 40 in Kompienga and 47 in Tapoa), all of them being infested by at least one tick. Four genera and six tick species were morphologically identified (Table 2). The most abundant species was *Amblyomma variegatum* (480/663; 72.4%), followed by *Hyalomma truncatum* (106/663; 16%) and *H. marginatum rufipes* (70/663, 10.6%). *Rhipicephalus lunulatus* and *R*. (*Boophilus*) *geigyi* were found only in Tapoa, while *R. sanguineus* was not collected in Gourma (Table 2). A total of 171 pools were established and tested for virus detection.

### 3.2. Viruses Detected in Ticks

Among the 171 pools, 24 pools (14%) were found positive for parapoxvirus genus-specific PCR. All other genus-specific PCR provided negative results. Of the total pools, 14/171 (8.2%) were positive for PCPV and 10/171 (5.8%) for BPSV based on their specific PCR and subsequent sequences analyses (Table 3). Out of the six tick species for which specimens were collected during this study, three species were found to be infected with PCPV: i.e., *A. variegatum* (7/14 pools), *H. m. rufipes* (4/14 pools), *H. truncatum* (3/14 pools), whereas two species were carrying BPSV: i.e., *A. variegatum* (9/10 pools) and *H. truncatum* (1/10 pools) (Table 4). The pools including other tick species were negative for both PCPV and BPSV. The infection rates for each virus species were not statistically significant within tick species (*p* > 0.05).

### 3.3. Phylogenetic Analysis

**Figure 2:**
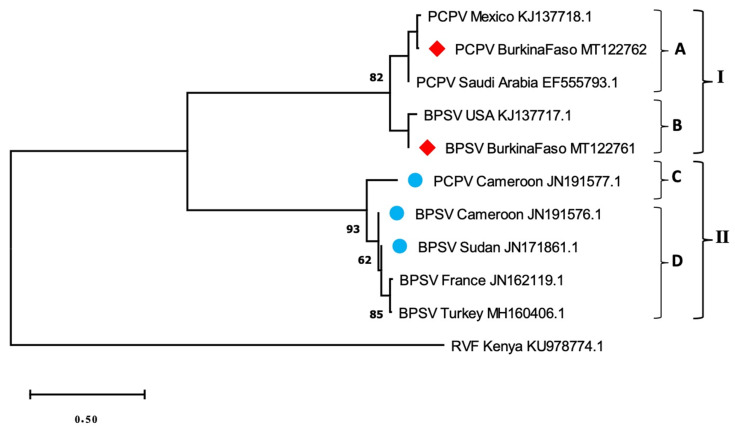
Neighbor-joining phylogenetic tree based on the partial sequences of the major envelope protein (B2L) gene of Bovine Papular Stomatitis and Pseudocowpox virus. Origins and accession numbers of reference sequences of BPSV (Bovine Papular Stomatitis Virus), PCPV (Pseudocowpox virus) as well as that of RVFV (Rift Valley Fever Virus) are indicated. Blue circles refer to samples from elsewhere in Africa, while red squares correspond to our samples from Burkina Faso.

**Figure 3:**
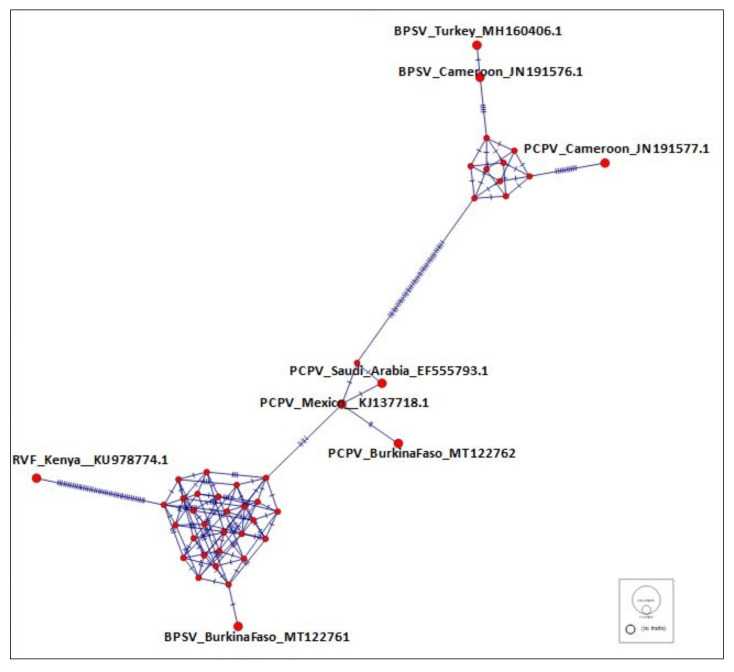
Reticulate tree, built with median joining network method.

The respective virus sequences were identical and only the longest sequence of each species was included in the phylogenetic analysis. The BPSV sequence obtained (MT122761) showed 100% identity, with a BPSV strain previously evidenced in the USA (KJ137717.1). As well, the closest strain of PCPV sample (MT122762) was a PCPV strain from Mexico (KJ137718.1), with 98.99% identity on 97% of the studied sequence. The neighbor-joining phylogenetic tree (Figure 2) showed a clustering of the sequences into two main groups, I and II, each containing the two viral species. The group I included subgroups A and B. The BPSV sequence from the present study gathered with a BPSV sample from USA within subgroup B. Meanwhile, the PCPV studied sequence was within the subgroup A, with other PCPV samples from Latin America and the Middle East (Figure 2). Group II comprised subgroups C and D and gathered reference samples from Europe and Africa (Figure 2). These gatherings were confirmed by a reticulate tree, built using the median joining network method in PopArt software (Figure 3).

## 4. Discussion

Several arboviruses have recently emerged and are now widespread across Africa, such as West Nile, Chikungunya and Zika viruses [18,20]. This region is now considered to contribute to the largest share of emerging vector-borne and zoonotic diseases [3]. Nevertheless, some areas are completely unexplored regarding these diseases, which is the case in Eastern Burkina Faso. The implementation of vectors and pathogens surveillance is facing many constraints, such as the limited resources and the unfavorable field conditions, with climatic and security factors being the most important limitations. The aim of the study was the screening of potential circulating arboviruses and the assessment of their zoonotic implications. Interestingly, no arbovirus was detected, despite the fact that adequate conditions for the preservation of the living ticks were strictly enforced. However, this study highlighted the presence of two parapoxviruses, PCPV and BPSV, in ticks collected from cattle in Eastern Burkina Faso. The tick species collected were expected to be found in the region, with *A. variegatum* and *Hyalomma* spp. being the most represented [19,21]. *A. variegatum* showed the highest detection rate for BPSV, while PCPV was found in the second most-represented species, *H. m. rufipes*. The natural interaction between ticks and parapoxvirus detected in our study remains unknown. The virus transmission most likely occurs by direct contact between infected and susceptible animals [10], although mechanical transfer by flies or ticks can probably occur as described for another poxvirus, the lumpy skin disease virus [11,22,23]. Domestic cattle are considered to be the main reservoir of both PCPV and BPSV, although buffalo were also reported to be a competent reservoir [24]. In this study, ticks collected in 3 herds of the Tapoa province were found positive for BPSV. It is noticeable that these herds were sharing water and pastures resources and that wildlife reserves are present in the area. Additionally, owners reported a history of seasonal transhumance via the same route. Taking into account the high environmental stability of poxvirus [25], common grazing that allows livestock to freely access natural resources shared with other herds and wildlife, the seasonal transhumance is likely to support the transmission of the virus. This could have important health and economic impacts, especially regarding transboundary cattle movements.

Besides their similarity to reference samples, studied samples seem to be closer to American samples than those from East and Central Africa. Such result suggests more gene flow between BPSV and PCPV from West Africa and America than with other regions. It could result from bovine importation from America to West Africa in order to improve local bovine production. For instance, Gir and Girolando were imported from Brazil to Benin and Côte d’Ivoire around 2002–2004 [26]. As well, the importation of Girolando from Brazil to Burkina Faso occurred in 1999. Moreover, although more analyses are needed to confirm some results of the current study, the differences between West African samples of BPSV and PCPV and those from East and Central Africa emphasize the pattern of cattle domestication in Africa [27].

Furthermore, it is noteworthy that the inspected cattle during field work were all asymptomatic, although the owners of positive herds described historical reports of clinical cases in cattle and humans (personal communication). It has been previously reported that parapoxvirus PCR positive can be identified from both symptomatic and asymptomatic ruminants [28]. Thus, it is likely that the sampled cattle were either healthy carriers of parapoxvirus and that the tick species found positive became infected through their blood meal, or that the ticks were already infected with parapoxviruses by feeding on domestic or wild animals before they clung to the exposed cattle. The lesions due to PCPV and BPSV in humans are often neglected and underreported by farmers. Although tick screening is not sufficient for the evaluation of the health status of the animal, this supports the relevance of the use of collected ticks for pathogens surveillance, especially for potential zoonotic diseases. Whether or not ticks play a role in the transmission of PCPV and/or BPSV remains to be studied, however, our finding represent preliminary data, serving as a basis for future studies. The cases of virus detection in vectors in West Africa remain poorly documented. The surveillance in animals and vectors may serve as an alert system to detect zoonotic arbovirus outbreaks such as Crimean Congo hemorrhagic fever, West Nile or Rift Valley fever viruses [18,28,29], and this is something which should be encouraged.

## Figures and Tables

**Table 1:** Primers and probes used for genus- and species-specific real-time PCR assays.

Genus or Species	Primer/Probe	5′→3′ Sequence	Target	Position	Amplicon Size (bp)	Concentration	Reference
Pan-Parapox viruses	Forward	TCGATGCGGTGCAGCAC	B2L	599–683	85	7.5 pmol	[16]
	Reverse	GCGGCGTATTCTTCTCGGAC				7.5 pmol	
	Probe	FAM-TGCGGTAGAAGCC-NFQ-MGB				2.5 pmol	
Pan-Parapox viruses	Forward	CGCGGTCTGGTCCTTG	J6R	771–855	85	0.4 µmol	[13]
	Reverse	CAGCATCAACCTCTCCTACATCA				0.4 µmol	
	Probe	FAM-CCACGAAGCTGCGCAGCAT-BHQ1				200 nmol/L	
Orf virus (ORF)	Forward	GAGTTCGAGGAGATGATCTTGA	ORFV_J6R	697–764	68	0.4 µmol	
	Reverse	FAM-GCCGAGGAGCAGGTCA				0.4 µmol	
	Probe	CTCGATCACGGCGCGCT-BHQ1				200 nmol/L	
Bovine papular stomatitis virus (BPSV)	Forward	GAGATGATCTTGATGTTGTCGTACT	BPSV_J6R	665–755	91	0.4 µmol	
	Reverse	FAM-TGGGCATGATCGTGAAGTAC				0.4 µmol	
	Probe	ATCATCGCGCGCTGGATCAC-BHQ1				200 nmol/L	
Pseudocowpox virus (PCPV)	Forward	CCGACTACATCCGGAACA	PCPV_J6R	62609–62675	67	0.4 µmol	
	Reverse	CGCACGCGCTTGCT				0.4 µmol	
	Probe	FAM-CTCACGCAGAAGATCTTCGTGAACTAC-BHQ1				200 nmol/L	
pan-Orthopox virus	OPE9L-F1880	GAA CAT TTT TGG CAG AGA GAG CC	HA (J7R)		177	0.5 µM	[17]
	OPE9L-R2057	CAA CTC TTA GCC GAA GCG TAT GAG				0.5 µM	
	OPE9L-p1924S-MGB	FAM-CAG GCT ACC AGT TCA A-MGBNFQ				0.1 µM	
Pan-Flaviviruses	PF1	TGYRTBTAYAACATGATGGG	NS5		93	20 µM	[18]
	PF2	GTGTCCCADCCDGCDGTRTC				20 µM	
Rift Valley Fever Virus	RVS	AAAGGAACAATGGACTCTGGTCA	G2	349–417	94	1 µM	[14]
	RVAs	CACTTCTTACTACCATGTCCTCCAAT				1 µM	
	RVP	AAAGCTTTGATATCTCTCAGTGCCCCAA				0.2 µM	
Crimean-Congo Hemorrhagic Fever Virus	RWCF	CAAGGGGTACCAAGAAAATGAAGAAGGC	S	1068–1223	181	600 nM	[15]
	RWCR	GCCACAGGGATTGTTCCAAAGCAGAC				600 nM	
	SE01	FAM-ATCTACATGCACCCTGCTGTGTTGACA-TAMRA				100 nM	
Pan-Phlebovirus	Phlebo forward 1	TTTGCTTATCAAGGATTTGATGC	N	210–400	370	50 pmol	[19]
	Phlebo forward 2	TTTGCTTATCAAGGATTTGACC				50 pmol	
	Phlebo reverse	TCAATCAGTCCAGCAAAGCTGGGATGCATCAT				50 pmol	

**Table 2:** Number of ticks collected in cattle in three provinces of eastern Burkina Faso.

Tick Species	Gourma	Kompienga	Tapoa	Total No. (%)
*A. variegatum*	6	222	252	480 (72.4)
*H. truncatum*	7	48	51	106 (16.0)
*H. m. rufipes*	9	23	38	70 (10.6)
*R. lunulatus*	--	--	3	3 (0.5)
*R. sanguineus*	--	1	1	2 (0.3)
*R. (B.) geigyi*	--	--	2	2 (0.3)
**Total No. (%)**	22 (3.3)	294 (44.3)	347 (52.3)	663

**Table 3:** Parapoxvirus detection in pools of ticks collected in each province of eastern Burkina Faso.

		*A. variegatum*	*H. m. rufipes*	*H. truncatum*	Total
**Gourma**	PCPV	0/4	2/9	0/6	2/19
	BPSV	1/4	0/9	0/6	1/19
**Kompienga**	PCPV	1/37	0/14	0/12	1/63
	BPSV	2/37	0/14	0/12	2/63
**Tapoa**	PCPV	6/46	2/20	3/19	11/85
	BPSV	6/46	0/20	1/19	7/85
**Total**	PCPV	7/87	4/43	3/37	14/167
	BPSV	9/87	0/43	1/37	10/167

**Table 4:** Positive tick species pools for bovine papular stomatitis virus (BPSV) and pseudocowpox virus (PCPV).

Pools ID	Tick Species	Farms ID	Cattle ID	Province	Virus Detected
24	*A. variegatum*	Gou13	12	Gourma	BPSV
12	*A. variegatum*	Kom09	72	Kompienga	BPSV
19	*A. variegatum*	Kom09	76	Kompienga	BPSV
4	*A. variegatum*	Tap09	75	Tapoa	BPSV
45	*A. variegatum*	Tap04	183	Tapoa	BPSV
78	*A. variegatum*	Tap08	335	Tapoa	BPSV
94	*A. variegatum*	Tap08	333	Tapoa	BPSV
180	*A. variegatum*	Tap08	334	Tapoa	BPSV
181	*A. variegatum*	Tap08	334	Tapoa	BPSV
81	*H. truncatum*	Tap08	335	Tapoa	BPSV
37	*H. m. rufipes*	Gou01	419	Gourma	PCPV
56	*H. m. rufipes*	Gou11	94	Gourma	PCPV
52	*A. variegatum*	Kom06	17	Kompienga	PCPV
83	*A. variegatum*	Tap06	306	Tapoa	PCPV
110	*A. variegatum*	Tap06	307	Tapoa	PCPV
58	*A. variegatum*	Tap06	309	Tapoa	PCPV
63	*A. variegatum*	Tap06	310	Tapoa	PCPV
64	*A. variegatum*	Tap06	310	Tapoa	PCPV
86	*H. truncatum*	Tap06	306	Tapoa	PCPV
65	*H. truncatum*	Tap06	310	Tapoa	PCPV
109	*H. m. rufipes*	Tap06	307	Tapoa	PCPV
66	*H. m. rufipes*	Tap06	310	Tapoa	PCPV
177	*A. variegatum*	Tap09	341	Tapoa	PCPV
176	*H. truncatum*	Tap09	341	Tapoa	PCPV

Gou: Gourma, Kom: Kompienga, Tap: Tapoa; A.: *Amblyomma*; H.: *Hyalomma*; H.m.: *Hyalomma marginatum.*
